# The dhikr and the mental health of the elderly in Aceh, Indonesia

**DOI:** 10.4102/hsag.v29i0.2456

**Published:** 2024-02-15

**Authors:** Sufyan Anwar, Siti M.F. Siregar, Teuku Alamsyah, Teuku Muliadi, Marniati Marniati, Khairunnas Khairunnas

**Affiliations:** 1 Faculty of Public Health, Universitas Teuku Umar, Aceh Barat, Indonesia

**Keywords:** aged, dhikr, mental well-being, remembrance, religious practice

## Abstract

**Background:**

The ageing population in Indonesia is increasing. However, biological and social changes and declines may trigger mental health problems among the elderly. Existing data show that the prevalence of mental health problems (depression) among the elderly in Indonesia is above 15%.

**Aim:**

This study aims to investigate the relationship between the *Majelis Zikir* (dhikr assembly) and the mental health of the elderly.

**Setting:**

The research was conducted in Aceh Barat District, Aceh Province, Indonesia.

**Methods:**

This study uses a retrospective cohort design. The study sample consists of individuals aged 60 years or older. The total sample size was 114 (57 exposed and 57 unexposed groups). The research instrument was in the form of a questionnaire.

**Results:**

The respondents had an average age of 67.61 years, with more females (63.2%), more educated (67.5%), more unemployed (71.1%) and 61.4% felt that their income was sufficient. The analysis showed that the relative risk value of members of the dhikr assembly was 1.857 (95% CI: 1.266–2.724) and showed a significant relationship between the dhikr assembly and mental health.

**Conclusion:**

Dhikr, as one of the forms of religious activities in Islam, has a positive role in older people’s mental health. Elderly members of the dhikr assembly are likelier to achieve better mental health.

**Contribution:**

The findings in this study can assist healthcare professionals and policymakers in their efforts to maintain and improve the mental health of the elderly through dhikr.

## Introduction

The elderly population (aged ≥ 60 years) continues to increase and is estimated to reach 22% by 2050 (Prihandini, Wardani & Tama 2022). The same condition is also happening in Indonesia. This is considered a positive impact on development through increased life expectancy. In response to this condition, the health world must be able to face the challenges of population structure changes, as it is understood that the elderly population undergoes several changes and declines in their biological, sociological and psychological aspects. The decreased organ function and immunity result in a more significant potential for them to suffer from various health problems, primarily degenerative diseases. Furthermore, the elderly population is often associated with increased social isolation and withdrawal, which can be considered a significant social problem (Datta et al. 2019; Quinn 2021). Changes and declines in biological aspects (such as chronic illnesses) and social aspects (such as ageism and loneliness) can trigger mental health problems among the elderly population (Pramesona & Taneepanichskul 2018; Ribeiro-Gonçalves, Costa & Leal 2023).

Mental health is defined ‘as a state of well-being in which every individual realises his or her potential, can cope with the normal stresses of life, can work productively and fruitfully, and can contribute to her or his community’ (WHO and Calouste Gulbenkian Foundation 2014). It is reported that mental or neurological disorders (excluding headache disorders) affect more than 20% of adults aged 60 years and over, where these disorders account for 6.6% of all disabilities (DALYs) in this age group (Kadariya, Gautam & Aro 2019). In America, it is estimated that around 11.4% of adults experience anxiety disorders, while about 6.8% experience mood disorders (Reynolds et al. 2015). Meanwhile, in Indonesia, referring to a study conducted by Handajani et al. (2022) on 4236 people aged 60 years or older, it is known that the prevalence of mental health problems (depression) is 16.3%. Furthermore, a survey involving 2818 older adults with diabetes in Indonesia showed a higher prevalence of mental health disorders, namely 19.3% (Azam et al. 2021). These two results provide an overview that the prevalence of mental health disorders in the elderly in Indonesia is above 15%, which is quite alarming. In addition, it is acknowledged that mental health disorders are responsible for poor health outcomes, premature death, human rights violations and economic losses nationally and globally. Individuals with mental health conditions often experience severe human rights violations, discrimination and stigma (Kadariya et al. 2019).

Several researchers have proven that many variables contribute to mental health problems in the elderly, including self-perceived health, social disruption, social support, family cohesiveness, resilience, quality of life, abuse, economic status and dependence on daily activities (Anwar et al. 2020; Anwar, Elida & Khairunnas 2022; Evandrou et al. 2017; Handajani et al. 2022; Motsamai & Mhaka-Mutepfa 2022; Wickramaratne et al. 2022). Additionally, researchers have also established a relationship between religious and spiritual activities and the mental health of the elderly (Leung & Pong 2021; Mahwati 2017). Generally, the positive impact of religious involvement on mental health is more prominent in people experiencing high levels of stress, such as older people, individuals with disabilities and those with medical issues (Moreira-Almeida, Neto & Koenig 2006).

Dhikr, a fundamental concept in Islam, entails the act of remembering or mentioning God, serving as a vital component of Islamic spirituality and daily devotion for Muslims (Quran 13:28). This practice involves the repetition of specific phrases or divine names, such as ‘SubhanAllah’ (Glory be to Allah) and ‘Allahu Akbar’ (Allah is the Greatest), aiming to establish an unceasing connection with the Divine, achieve spiritual purification and draw nearer to Allah (Hadith, Sahih Muslim). Dhikr offers numerous spiritual benefits, including inner peace, forgiveness of sins and increased mindfulness of God, making it a central element in the spiritual lives of Muslims worldwide. Whether performed individually or collectively, dhikr remains a cornerstone of Islamic devotion and serves as a means of expressing gratitude and seeking spiritual enlightenment (Febrianty, Dinata & Jafari 2023).

Dhikr (remembrance of God) is a recommended religious ritual among Muslims. Positive feelings and self-confidence can grow through dhikr, which can reduce and eliminate anxiety and improve mental (Burhanuddin 2020). Dhikr can be performed individually or collectively in a group or majlis (assembly). People can perform it while standing, sitting or lying down. Performing it in a group allows people in the dhikr assembly to interact before and after the activity. This condition may positively impact the emotional and social relationship quality of the members of the dhikr assembly, which can improve mental health. Therefore, this study aims to assess the relationship between membership status in the dhikr assembly and mental health among the elderly population.

## Materials and methods

### Study design

This study was an observational study using a retrospective cohort design. Observations in retrospective cohort studies were made on the effect that has already occurred, while past risk factors were obtained through historical records. The research subjects were divided into two groups, the exposed group and the non-exposed group. Subjects who participated in the dhikr assembly were included in the sensitive group, while those who did not participate in the dhikr assembly were included in the non-exposed group.

### Study population

The study sample involved individuals aged 60 years or older, as at this age, many older adults tend to experience mental health problems. The following criteria must be met to become a research sample:

Inclusion criteria:

1. Subject age was 60 years or older at the time of the study.
2. Being able to communicate.
3. Have participated in the dhikr assembly for at least 3 months for the exposed group.
4. Willing to be a respondent by signing a statement letter.

Exclusion criteria:

1. Severe illness during the study, making data collection impossible.
2. Withdrawing or requesting to stop during the study or interview.

The formula used to determine the study’s sample size was the cohort study sample size calculation formula recommended by Kuntoro (2015). With α = 0.05, β = 0.10 and relative risk (RR) = 2.42, the sample size for this study was known to be 57 members of the dhikr assembly and 57 non-members of the dhikr gathering. In total, the sample size of the study was 114. The sample selection was conducted randomly in three subdistricts (Kaway XVI, Meureubo and Pante Cermin) in Aceh Barat District. The reason was that these three subdistricts have more members of the dhikr assembly and regularly perform dhikr activities. This dhikr assembly is under one organisation named the *Majelis Pengkajian Tauhid Tasauf* (*MPTT*).

### Research instrument

Keyes (2018) developed a questionnaire called the Mental Health Continuum Short Form (MHC-SF) to measure mental health. The questionnaire has three dimensions, including emotional, social and psychological well-being. The total number of questions is 14, including 3 about emotional well-being, 5 about social well-being and 6 about psychological well-being. The assessment uses a Likert scale, which evaluates the respondent’s condition regarding all three aspects in the past month. The answer choices for each question are ‘never; once or twice; once a week; two or three times a week; almost every day; and every day’. The original questionnaire is in English, but it was translated into Indonesian before being used for data collection. The assessment of the dhikr assembly uses one question, ‘Are you a member of the dhikr assembly?’ and respondents are asked to answer ‘yes’ or ‘no’.

### Statistical analysis

A non-parametric analysis, specifically the chi-square test, was employed to address the research objectives. In order to streamline the analytical process, the data on dhikr assembly membership status were categorised into two distinct groups, serving as the dependent variable: ‘yes’ denoted respondents who were members of the dhikr assembly, while ‘no’ indicated non-members. Meanwhile, mental health, serving as the independent variable, was also categorised into two groups. The reference point for this categorisation was the average total score value of all respondents in comparison to the individual respondent’s score. If a respondent’s score fell less than or equal to the average total score, they were classified as having low mental health. Conversely, if a respondent scored above the average total score, they were categorised as older adults with high or better mental health. This categorisation method was also applied to group the three dimensions of mental health, where each respondent’s score for a particular dimension was compared to the average total score of all respondents in that dimension.

This approach to data analysis was chosen for its suitability in handling categorical data, such as membership status in the dhikr assembly and mental health categories. The chi-square test was employed to assess any statistically significant associations or differences between these categories. It allowed for a comprehensive examination of the relationship between dhikr assembly membership and mental health, providing valuable insights into the impact of religious and spiritual activities on the mental well-being of older adults.

### Ethical considerations

The research permit for this study has been submitted to and approved by the Ethics Commission. The approval ensures that the study adheres to ethical standards and guidelines in conducting research involving human subjects. The study adheres to ethical principles, including respecting individual rights, promoting the greater good, and ensuring fairness. Before data collection, the study’s objectives were clearly explained to each participant. Participants were given freedom and the right to participate voluntarily and withdraw without consequences. Participants who agreed to participate in the study were asked to sign a written consent form provided by the researcher. The identity or name of the participants was not disclosed during data collection. This was done to protect and maintain the confidentiality of the participants, especially about the data they provided. Ethical approval 2622/VIII/SP/2022 granted by the Health Research Ethics Committee of the Faculty of Nursing at Universitas Sumatera Utara.

## Results

Table 1 shows that the total number of respondents is 114, with 57 respondents being members of the dhikr assembly and 57 respondents not being members of the dhikr assembly. The average age of the dhikr assembly group is 66.39 years (s.d.: 6.72), while the average age of the non-dhikr assembly group is 68.84 years (s.d.: 9.43). Furthermore, both among the dhikr assembly and non-dhikr assembly groups, there are more individuals in the 60–69 age range, accounting for 71.9% and 57.9%, respectively. In the dhikr assembly group, there is a higher percentage of female respondents (59.6%), more individuals who have received formal education (73.4%), more individuals who are no longer employed (64.9%) and more individuals who perceive their income as sufficient (64.9%). Similarly, in the non-dhikr assembly group, the data show a higher percentage of female respondents (66.7%), more individuals who have received formal education (61.4%), more individuals who are not employed (77.2%) and more individuals who perceive their income as sufficient (57.9%).

**TABLE 1:** Description of elderly sociodemographic characteristics.

Sociodemographic characteristics	The members of the dhikr assembly (*N* = 57)			Not the members of the dhikr assembly (*N* = 57)		
	F	Mean (%)	s.d.	F	Mean (%)	s.d.
**Age (years):**	-	66.39	6.72	-	68.84	9.43
60–69	41	71.90	-	33	57.90	-
≥70	16	28.10	-	24	42.10	-
**Sex:**	-	-	-	-	-	-
Men	23	40.40	-	19	33.30	-
Women	34	59.60	-	38	66.70	-
**Education:**	-	-	-	-	-	-
No school	15	26.30	-	22	38.60	-
School	42	73.40	-	35	61.40	-
**Job status:**	-	-	-	-	-	-
Work	20	35.10	-	13	22.80	-
Doesn’t work	37	64.90	-	44	77.20	-
**Income:**	-	-	-	-	-	-
Enough	37	64.90	-	33	57.90	-
Not enough	20	35.10	-	24	42.10	-

s.d., standard deviation.

It has been previously stated that this study uses a retrospective cohort design, so statistical testing only focuses on one independent variable (the dhikr assembly) and its relationship with mental health, including its dimensions. The analysis of the relationship between the dhikr assembly and each size of mental health (Table 2) shows that the dhikr assembly is significantly associated with older people’s emotional and social well-being, where the *p*-value is <0.05. In contrast, the dhikr assembly and psychological well-being are not significantly associated (*p* > 0.05). Nevertheless, all RR values indicate above 1.5 times, namely dhikr and emotional well-being (RR = 1.625), dhikr and social well-being (RR = 1.8018) and dhikr and psychological well-being (RR = 1.524). These results indicate that older people who are members of the dhikr assembly are 1.6 times more likely to achieve better emotional well-being than those who are not. Furthermore, older people who are members of the dhikr assembly are 1.8 times more likely to perform better social well-being than those who are not. Lastly, older people who are members of the dhikr assembly have 1.5 times potential to achieve better psychological well-being than those who are not.

**TABLE 2:** Distribution based on dimensions of mental health (emotional, social and psychological well-being) and dhikr assembly.

Variables	High		Low		Total	*p*	RR	95% CI	
	*N*	%	*N*	%				Lower	Upper
**Emotional well-being**									
Dhikr assembly									
Yes	39	68.4	18	31.6	57	0.008*	1.625	1.143	2.310
No	23	42.1	33	57.9	57	-	-	-	-
**Social well-being**									
Dhikr assembly									
Yes	40	70.2	17	29.8	57	0.001*	1.818	1.258	2.629
No	22	38.6	35	61.4	57	-	-	-	-
**Psychological well-being**									
Dhikr assembly									
Yes	32	56.1	25	43.9	57	0.060	1.524	1.011	2.296
No	21	36.8	36	63.2	57	-	-	-	-

* Significant *p*-value; RR, relative risk.

Table 3 presents the statistical analysis results of the association between the dhikr assembly and mental health, a combination of the three dimensions mentioned in Table 2. The study showed a *p*-value of 0.001 for the relationship between the dhikr assembly and psychological well-being, with an RR value of 1.857 (95% CI: 1.266–2.724). The *p*-value indicates a significant relationship between the dhikr assembly and the psychological well-being of older people. Meanwhile, the RR value suggests that older people who participate in or become members of the dhikr assembly have a 1.8 times higher chance of achieving better mental health than those who do not participate in the dhikr assembly.

**TABLE 3:** Distribution of dhikr assembly and mental health in older people.

Variables	Mental Health				Total	*p*	RR	95% CI	
	High		Low					Lower	Upper
	*N*	%	*N*	%					
Dhikr assembly									
Yes	39	68.4	18	31.6	57	0.001*	1.857	1.266	2.724
No	21	36.8	36	63.2	57	-	-	-	-

* Significant *p*-value; RR, relative risk.

## Discussion

Religion is an organised system of beliefs, ritual practices and symbols designed to facilitate closeness with the sacred or transcendent (God, higher power or ultimate truth or reality) (Lucchetti, Koenig & Lucchetti 2021; Moreira-Almeida, Neto & Koenig 2006). Rituals or worship in Islam encompass all human activities performed with a spiritual attitude and intention to devote or surrender oneself to Allah SWT (Hidayat 2017). Rituals cannot be separated from beliefs because the mood and sense of a person’s worship expressed in the form of rituals are based on their ideas. Rituals are considered evidence of human thought in the existence of a mighty power beyond human capability. Some rituals in Islam have specific provisions in their implementation, and they are all contained in the holy texts. In addition to being a form of devotion, Choellho-Júnior et al. explain that participation and obedience in religious ritual practices can impact various parameters related to health, including mental health (2022). Religious rituals can positively benefit mental health, such as reducing anxiety, providing meaning and purpose and fostering a sense of community ownership (Coppola et al. 2021). Therefore, active participation of the elderly in religious ritual activities such as dhikr is highly recommended, as, in general, the elderly are very vulnerable to mental health issues (Coelho-Júnior et al. 2022).

Our study results indicated that the dhikr assembly was significantly associated with mental health among older people (*p* = 0.001). The relationship between the two was positive. Older people who were members of the dhikr assembly had better mental health conditions and a greater chance of achieving better mental health, 1.8 times more than those who were not. These findings indicate that the recognition of the existence and power of Allah through dhikr may be negatively associated with mental health problems such as psychological problems, anxiety, depression, emotional disorders and social relationship problems among the elderly. Tangsangwornthamma states that dhikr can overcome negative feelings and support positive outcomes in those who practice it (Tangsangwornthamma, Ahmad & Rattanamongkolgul 2018). Dhikr is even considered a highly effective way of combating depression in individuals (Abdullah & Zaki 2020). Positive psychological impacts will be felt when a person performs dhikr with sincere feelings (Abdullah et al. 2022). These study findings were supported by several previous studies that have shown a relationship between dhikr and mental health, such as Tangsangwornthamma et al. in Thailand (2018), Abdullah and Zaki (2020) and Mansor, Yassin and Ahmad (2020) in Malaysia. Several studies in Indonesia also showed significant results on the relationship between dhikr and mental health. Some of them are dhikr training studies and anxiety reduction in elderly hypertensive patients (Binoriang & Pramesti 2021), dhikr therapy and music therapy with a decrease in depression levels in the elderly (Bahtiar, Sahar & Widyatuti 2020) and dhikr treatment and a decrease in anxiety levels in older adults with cognitive function disorders (Agustina, Handayani & Nurjanah 2020; Juniarni, Putri & Rachma 2022).

Ritual dhikr is a medium for Muslims to remember Allah SWT. The soul’s attachment to Allah SWT is formed when a person does dhikr, creating feelings of love for Allah (Abdullah et al. 2022). During dhikr, the practitioner invites their tongue to speak, ears to hear, eyes to imagine and mind to concentrate on specific phrases about Allah’s greatness and glory, such as ‘Astaghfirullahal azim, Subhanallah, Alhamdulillah, Allahu akbar, La ilaha illallah’. According to Solihin’s statement, textual and contextual similarities must be fostered in implementing rituals, namely the word ‘Allah’ (Hidayat 2017). Dhikr can be recited silently or aloud and is considered a form of meditation for Muslims (Syed 2003), as it is not too different from meditation in Buddhism, Christianity and Hinduism (Hamsyah & Subandi 2016), especially if done silently (*sir*).

Dhikr is more focused on the heart or *qalb* when practicing remembrance (Zuhri, Anwar & Marzuki 2020) and constantly mentioning Allah’s name while including the mind. The aim is to achieve a state of consciousness and closeness to Allah SWT. Dhikr is a verbal utterance of Allah’s name and a heart training always to be aware of Allah’s presence and seek His pleasure in all aspects of life. This process purifies the soul and removes negative thoughts and emotions, replaced by positive feelings such as gratitude, love and satisfaction. Through dhikr, a person strives to become a better individual who is more aware of Allah’s blessings and more committed to following His guidance. Thus, dhikr is a method of instilling the noble qualities of Allah SWT because remembering Allah SWT through dhikr can help improve an individual’s behaviour by strengthening their faith values (Abdullah et al. 2022). Hamsyah and Subandi explained several positive impacts that may arise in individuals through dhikr (Hamsyah & Subandi 2016). Firstly, performing dhikr helps to spiritually and psychologically cleanse oneself. Individuals who perform dhikr feel that all their burdens of thought are lifted. They will have an optimistic personality and have positive expectations when facing problems in life. Secondly, after performing the dhikr ritual, people feel closer to Allah. This closeness brings love, happiness, pleasure and other positive emotions. Thirdly, individuals who perform dhikr lose their ego and fully surrender themselves to Allah. Arrogance and haughtiness will disappear with dhikr (Mustaqim 2015), thus increasing gratitude for everything in their lives. Lastly, performing dhikr helps individuals find meaning in their daily lives. Seeking life’s purpose increases satisfaction because they gain wisdom from everything that happens in life.

This study focuses on the dhikr assembly managed by the MPTT because it has a management and an organisational structure. In an area consisting of several villages, assembly members build a place or hall of dhikr. According to the agreed schedule, Dhikr is routinely performed at the entrance at least once weekly. Although members come from various age groups, there are more adults and older adults. They come from multiple villages or regions, social classes and groups. Performing dhikr starts with intention, then reciting istighfar (seeking forgiveness from Allah SWT) and salawat (sending greetings to Prophet Muhammad) approximately three times. The next activity is reading Quran; *Surah Al Fatihah* approximately once and then entering the core activity of dhikr by saying ‘La ilaha illallah’ about 1000 times or as much as possible. Dhikr activities are usually concluded with prayer. Dhikr is done sitting and separated between male and female congregations, even though they are in the same hall.

In more detail, Nisma explained several other characteristics of this dhikr activity as follows (Nisma 2020): firstly, dhikr is done collectively by loudly saying ‘lailahaillallah’ (*jihar*). This creates social contact among members before and after dhikr, which may impact the social well-being of older people. Secondly, the male congregation turns off lights to enhance concentration. Thirdly, wearing white clothing during dhikr symbolises purity of the heart. Purity of the heart and peace of mind are also considered the goal of dhikr, where negative traits such as envy, jealousy, impatience and insincerity should be eliminated. These traits hurt a person’s psychological state. Through consistent dhikr, several spiritual diseases will slowly erode and disappear within a person. Allah SWT explained in the Quran; *Surah Ar-Ra’d* verse 28, which means, ‘Those who have believed and whose hearts are assured by the remembrance of Allah. Unquestionably, by the memory of Allah, hearts are assured’ (Zuhri, Anwar & Marzuki 2020).

This study suggests that every elderly Muslim should participate in religious activities, especially collectively, such as the dhikr assembly. Dhikr can bring one closer to Allah and is a form of the human relationship with the Creator (*hablumminallah*). Older people also have the opportunity to interact socially with other members of the majlis as a form of human connection with others (*hablumminannas*). Policymakers and healthcare professionals need to consider efforts to improve older people’s mental health through religious practices such as the dhikr assembly. They need to encourage the continuation and active participation in the dhikr assembly. In addition to directly impacting mental health, the dhikr assembly provides a platform for healthcare professionals to empower older people or convey health messages. Therefore, collaboration and coordination must be developed with religious figures or organisations, especially those with the dhikr assembly. However, this study has limitations, including the design approach≈used (retrospective cohort) and the sample size involved in the study. Research with different designs, including prospective affiliates and experiments with higher-quality studies, must be conducted to strengthen these findings. Furthermore, this study only took samples of older adults in Aceh Barat Regency, so it does not represent nationally generalisable results in Indonesia. Therefore, a more prominent and representative sample in the following study must be considered, given that most Indonesian population, including the elderly, is Muslim.

## Conclusion

In-depth analysis of the study’s findings underscores the significant role of the dhikr assembly in enhancing the mental health of older individuals. The research reveals that older adults who actively participate in the dhikr assembly experience a substantial 1.8-fold increase in their likelihood of achieving superior mental well-being compared to their non-participating counterparts. This empirical evidence carries important implications for healthcare professionals, as it highlights the potential for implementing targeted strategies to promote mental health among the elderly through their engagement in the dhikr assembly. Furthermore, recognising the dhikr assembly as a platform, it becomes evident that it can serve as an effective conduit for conveying valuable health-related messages to the broader elderly population, extending its impact beyond individual well-being to the wider community.

## References

[CIT0001] Abdullah, S., Kastriafuddin, T., Tengku S, Mahamood, A.F., Hussin, N.S., Mahmad, M.A. et al., 2022, ‘The relationship between religious coping and Islamic Mental Health personality during the Covid-19 pandemic’, *Journal of Pharmaceutical Negative Results* 13(10), 4290–4298. 10.47750/pnr.2022.13.S10.519

[CIT0002] Abdullah, W.H.W. & Zaki, H., 2020, ‘Depression symptoms: Methods of treatment through al-Tibb al-Nabawi medicine’, *Jurnal Islam dan Masyarakat Kontemporari* 21(3), 215–234. 10.37231/jimk.2020.21.3.509

[CIT0003] Agustina, N.W., Handayani, S. & Nurjanah, L., 2020, ‘Effects of reading dhikr Asmaul Husna Ya Rahman and Ya Rahim against changes in the level of anxiety in the elderly’, *Journal of Physics: Conference Series* 1517(1), 012049. 10.1088/1742-6596/1517/1/012049

[CIT0004] Anwar, S., Supriyanto, S., Budiarto, W., & Hargono, R., 2020, ‘Relationship between social capital and mental health among the older adults in Aceh, Indonesia’, *Indian Journal of Forensic Medicine & Toxicology* 14(3), 2204–2209. 10.37506/ijfmt.v14i3.10755

[CIT0005] Anwar, S., Elida, S., & Khairunnas. 2022, ‘The contribution of family cohesion and self-efficacy on the mental health of older adults: A survey conducted in Aceh, Indonesia’, *Indian Journal of Forensic Medicine & Toxicology* 16(2), 387–394. 10.37506/ijfmt.v16i2.17998

[CIT0006] Azam, M., Sulistiana, R., Fibriana, A.I., Savitri, S. & Aljunid, S.M., 2021, ‘Prevalence of mental health disorders among elderly diabetics and associated risk factors in Indonesia’, *International Journal of Environmental Research and Public Health* 18(10301), 1–2. 10.3390/ijerph181910301 PMC8508332 34639601

[CIT0007] Bahtiar, B., Sahar, J. & Widyatuti, W., 2020, ‘Music, dhikr, and deep breathing technique to decrease depression level in older adults: Evidence-based practice in Depok City, Indonesia’, *ASEAN Journal of Community Engagement* 4(2), 468–484. 10.7454/ajce.v4i2.1093

[CIT0008] Binoriang, D.P. & Pramesti, S.W., 2021, ‘The comparison of the effectiveness between cananga aromatherapy and dzikr therapy on reducing anxiety in the elderly with hypertension at posyandu Tawarsari Wonosari Gunungkidul’, *Bali Medical Journal* 10(3), 1263–1267. 10.15562/bmj.v10i3.2871

[CIT0009] Binoriang, D.P., & Pramesti, S.W., 2021, ‘The comparison of the effectiveness between cananga aromatherapy and dzikr therapy on reducing anxiety in the elderly with hypertension at posyandu Tawarsari Wonosari Gunungkidul’, *Bali Medical Journal*, 10(3), pp. 1263–1267. 10.15562/bmj.v10i3.2871.

[CIT0010] Burhanuddin, B., 2020, ‘Zikir Dan Ketenangan Jiwa (Solusi Islam Mengatasi Kegelisahan dan Kegalauan Jiwa)’, *Jurnal Mimbar: Media Intelektual Muslim dan Bimbingan Rohani* 6(1), 15–25. 10.47435/mimbar.v6i1.371

[CIT0011] Coelho-Júnior, H.J., Calvani, R., Panza, F., Allegri, R.F., Picca, A., Marzetti, E. et al., 2022, ‘Religiosity/spirituality and mental health in older adults: A systematic review and meta-analysis of observational studies’, *Frontiers in Medicine* 9, 877213. 10.3389/fmed.2022.877213 35646998 PMC9133607

[CIT0012] Coppola, I., Rania, N., Parisi, R. & Lagomarsino, F., 2021, ‘Spiritual well-being and mental health during the COVID-19 pandemic in Italy’, *Frontiers in Psychiatry* 12, 626944. 10.3389/fpsyt.2021.626944 33868047 PMC8046904

[CIT0013] Datta, A., Bhatia, V., Noll, J. & Dixit, S., 2019, ‘Bridging the digital divide: Challenges in opening the digital world to the elderly, poor, and digitally illiterate’, *IEEE Consumer Electronics Magazine* 8(1), 78–81. 10.1109/MCE.2018.2867985

[CIT0014] Evandrou, M., Falkingham, J.C., Qin, M. & Vlachantoni, A., 2017, ‘Elder abuse as a risk factor for psychological distress among older adults in India: A cross-sectional study’, *BMJ Open* 7(10), e017152. 10.1136/bmjopen-2017-017152 PMC5665217 29061615

[CIT0015] Febrianty, A., Dinata, K.I. & Jafari, M., 2023, ‘A dhikr media approach to achieve a healthy soul’, *International Conference on Tradition and Religious Studies* 2(1), 100–109.

[CIT0016] Hamsyah, F. & Subandi, 2016, ‘Dzikir and happiness: A mental health study on an Indonesian Muslim Sufi Group’, *Journal of Spirituality in Mental Health* 19(1), 80–94. 10.1080/19349637.2016.1193404

[CIT0017] Handajani, Y.S., Schröder-Butterfill, E., Hogervorst, E., Turana, Y. & Hengky, A., 2022, ‘Depression among older adults in Indonesia: Prevalence, role of chronic conditions and other associated factors’, *Clinical Practice & Epidemiology in Mental Health* 18(1), e174501792207010. 10.2174/17450179-v18-e2207010 37274861 PMC10156049

[CIT0018] Hidayat, M.A., 2017, ‘Worship, the body and identity: Islamic rituals and the construction of Muslim identity’, *The Journal of Society and Media* 1(2), 1–17. 10.26740/jsm.v1n2.p1-17

[CIT0019] Juniarni, L., Putri, T.A.R.K. & Rachma, A., 2022, ‘The efficacy of dhikr therapy on anxiety in the elderly people with decreased cognitive function’, *Malaysian Journal of Medicine and Health Sciences* 18(Supp 17), 139–146.

[CIT0020] Kadariya, S., Gautam, R., & Aro, A.R. 2019, ‘Physical Activity, Mental Health, and Wellbeing among Older Adults in South and Southeast Asia: A Scoping Review’, *BioMed research international*, 2019, 6752182. 10.1155/2019/6752182. 31886239 PMC6925721

[CIT0021] Keyes, C., 2018, *Overview of The Mental Health Continuum Short Form (MHC-SF)*, Emory University, Atlanta. 10.13140/RG.2.2.24204.62088

[CIT0022] Kuntoro, H., 2015, *Metode sampling dan penentuan besar sampel*, Pustaka Melati, Surabaya.

[CIT0023] Leung, C.H. & Pong, H.K., 2021, ‘Cross-sectional study of the relationship between the spiritual wellbeing and psychological health among university Students’, *PLoS One* 16(4), e0249702. 10.1371/journal.pone.0249702 33857211 PMC8049307

[CIT0024] Lucchetti, G., Koenig, H.G. & Lucchetti, A.L.G., 2021, ‘Spirituality, religiousness, and mental health: A review of the current scientific evidence’, *World Journal of Clinical Cases* 9(26), 7620–7631. 10.12998/wjcc.v9.i26.7620 34621814 PMC8462234

[CIT0025] Mahwati, Y., 2017, ‘The relationship between spirituality and depression among the elderly in Indonesia’, *Makara Journal of Health Research* 21(1), 13–19. 10.7454/msk.v21i1.6206

[CIT0026] Mansor, A.B.A., Yassin, K.M. & Ahmad, S., 2020, ‘Analysis of qodiriyah naqsyabandiyah’s zikir tariqat as a therapy for drug recovery in Malaysia’, *Journal of Critical Reviews* 7(8), 1580–1585. 10.31838/jcr.07.08.312

[CIT0027] Moreira-Almeida, A., Neto, F.L. & Koenig, H.G., 2006, ‘Religiousness and mental health: A review’, *Rev Bras Psiquiatr* 28(3), 242–250. 10.1590/S1516-44462006005000006 16924349

[CIT0028] Motsamai, T.B. & Mhaka-Mutepfa, M., 2022, ‘Depression: Determinants that influence the mental health of older people (60 years +) in Botswana’, *Gerontology and Geriatric Medicine* 8, 233372142110531. 10.1177/23337214211053121 PMC8883394 35237710

[CIT0029] Mustaqim, 2015, ‘Pemikiran Islam Kontemporer: Konsep Dhikr Allah dan Urgensitasnya dalam Masyarakat’, *Al Mabsut: Jurnal Studi Islam dan Sosial* 9, 77–105.

[CIT0030] Nisma, Y., 2020, ‘Rateb Siribee: Spiritualitas dan Solidaritas Religius Masyarakat Pedesaan Aceh’, *Jurnal Sosiologi Agama Indonesia* 1, 32–48. 10.22373/jsai.v1i1.423

[CIT0031] Pramesona, B.A. & Taneepanichskul, S., 2018, ‘Prevalence and risk factors of depression among Indonesian elderly: A nursing home-based cross-sectional study’, *Neurology, Psychiatry and Brain Research* 30, 22–27. 10.1016/j.npbr.2018.04.004

[CIT0032] Prihandini, N., Wardani, H.E. & Tama, T.D., 2022, ‘Early detection and determinants of dementia in the working area of Mojolangu Public Health Center, Malang (Indonesia)’, *Journal of Public Health in Africa* 13(suppl. 2), 2410. 10.4081/jphia.2022.2410 37497129 PMC10367027

[CIT0033] Quinn, K., 2021, ‘Social media and social wellbeing in later life’, *Ageing and Society* 41(6), 1349–1370. 10.1017/S0144686X19001570 37034465 PMC10081153

[CIT0034] Reynolds, K., Pietrzak, R.H., El-Gabalawy, R., Mackenzie, C.S. & Sareen, J., 2015, ‘Prevalence of psychiatric disorders in U.S. older adults: Findings from a nationally representative survey’, *World Psychiatry* 14(1), 74–81. 10.1002/wps.20193 25655161 PMC4329900

[CIT0035] Ribeiro-Gonçalves, J.A., Costa, P.A. & Leal, I., 2023, ‘Loneliness, ageism, and mental health: The buffering role of resilience in seniors’, *International Journal of Clinical and Health Psychology* 23(1), 100339. 10.1016/j.ijchp.2022.100339 36168598 PMC9485034

[CIT0036] Syed, I.B., 2003, ‘Spiritual medicine in the history of Islamic medicine’, Journal of the International Society for the History of Islamic Medicine 2(1), 45–49.

[CIT0037] Tangsangwornthamma, C., Ahmad, N. & Rattanamongkolgul, S., 2018, ‘A qualitative study on belief, perception, and health effects on standing Zikr among Thai Muslims in Nakhon Nayok Province’, *Journal of Psychology Research* 8(11),544–557. 10.17265/2159-5542/2018.11.002

[CIT0038] Wickramaratne, P.J., Yangchen, T., Lepow, L., Patra, B.G., Glicksburg, B., Talati, A. et al., 2022, ‘Social connectedness as a determinant of mental health: A scoping review’, *PLoS One*, 17(10), e0275004. 10.1371/journal.pone.0275004 36228007 PMC9560615

[CIT0039] World Health Organization and Calouste Gulbenkian Foundation, 2014, *Social determinants of mental health*, vol. 106, pp. 20–27, World Health Organization, Geneva.

[CIT0040] Zuhri, A., Anwar, H. & Marzuki, M., 2020, ‘The Remembrance Concept as a Medium of Quality Soul’, *Al-Hikmah Journal of Theosophy and Islamic Civilization* 2(1), 39–65.

